# Supplementary material for: SAAINT-DB: a comprehensive structural antibody database for antibody modeling and design
Source: Acta Pharmacol Sin. 2025 Jun 30;46(12):3365–75. doi: 10.1038/s41401-025-01608-5 (PMC12644554; doi:10.1038/s41401-025-01608-5)
Supplement: Supplementary file 1 — Supplemenraty information [file 41401_2025_1608_MOESM1_ESM.pdf]

Supplementary information

SAAINT-DB: a comprehensive structural antibody database for antibody modeling and design

Xiaoqiang Huang[1]*, Jun Zhou[1], Shuang Chen[1], Xiaofeng Xia[2], Y. Eugene Chen[1], Jie Xu[1]

[1]Center for Advanced Models for Translational Sciences and Therapeutics, University of Michigan Medical School, 2800 Plymouth Road, Ann Arbor, MI 48109, USA

[2]Research & Development, ATGC Inc., 1004 W 9th Avenue, King of Prussia, PA 19406, USA

*Correspondence: xiaoqiah@umich.edu

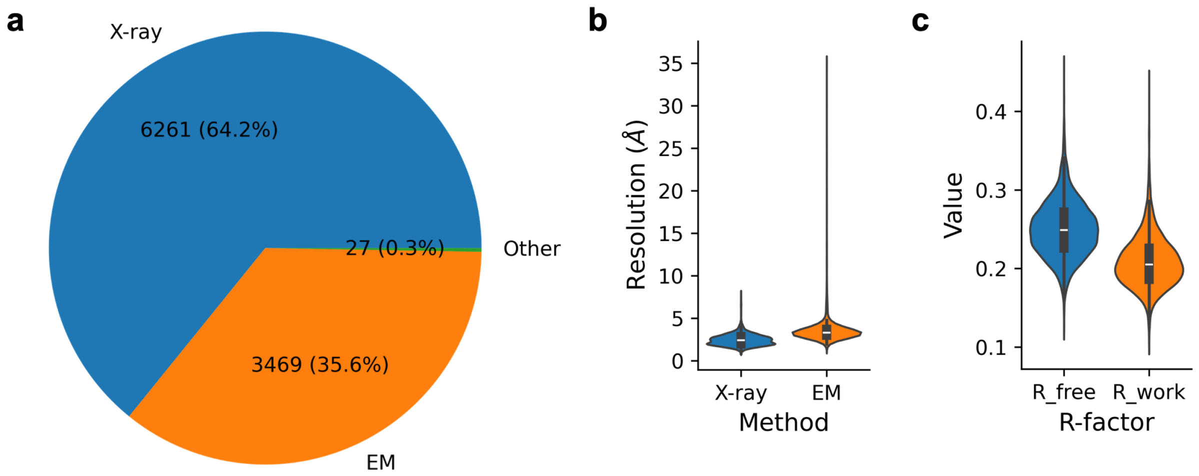

Supplementary Fig. 1. Statistics of antibody structure determination methods and structural quality in SAAINT-DB. a, Distribution of experimental methods for solving PDB structures in SAAINT-DB. **b**, Distribution of resolution for X-ray and electron microscopy (EM) structures. **c**, Distribution of R-factor values for X-ray structures.

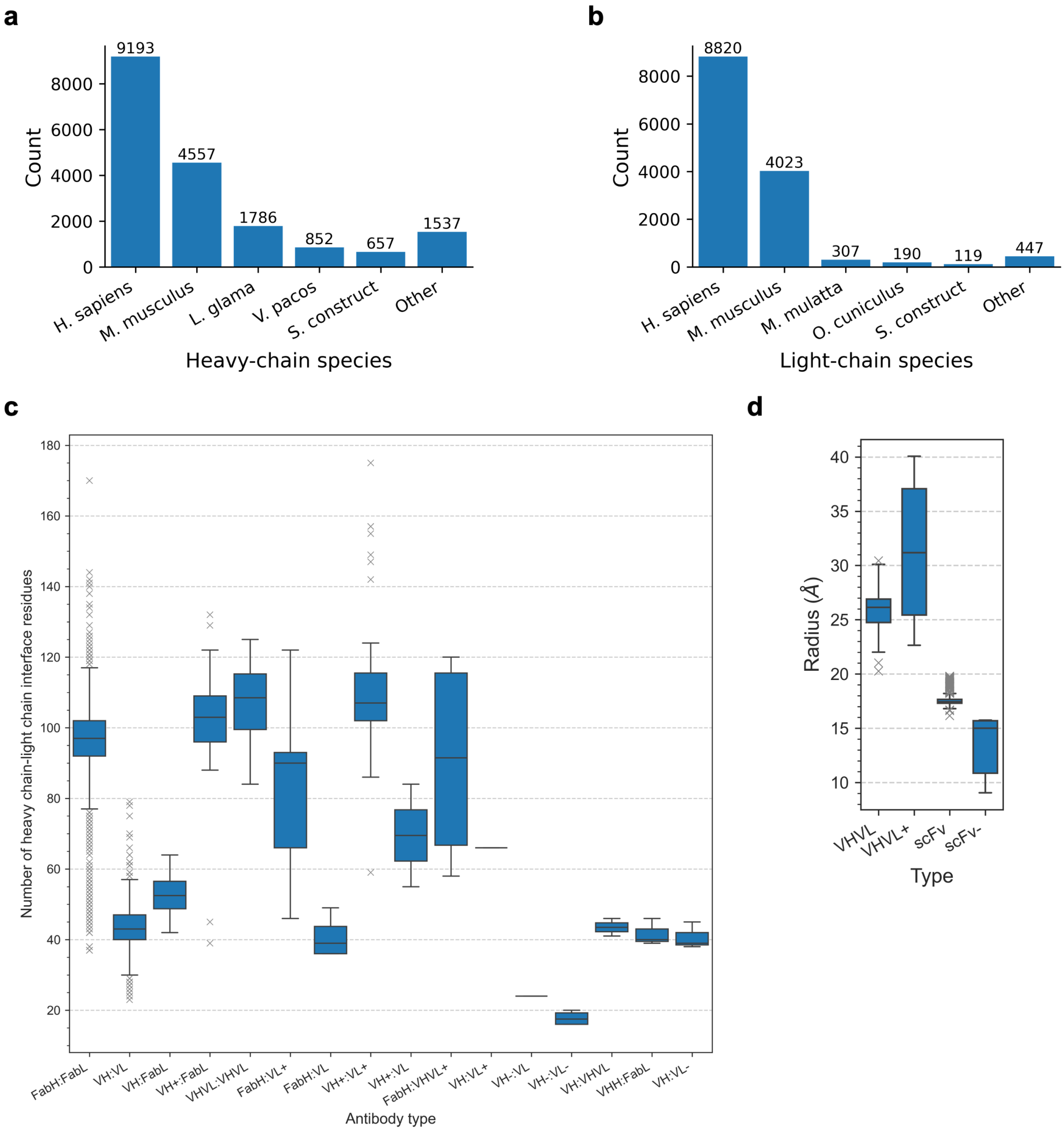

Supplementary Fig. 2. Additional statistics on antibodies in SAAINT-DB. a, Top sources of antibody heavy chains. **b**, Top sources of antibody light chains. **c**, Distribution of the number of heavy chain-light chain interface residues across distinct antibody types. **d**, Distribution of the mean radius for scFv and VHVL types. See the main text for the definition of antibody chain mean radius.

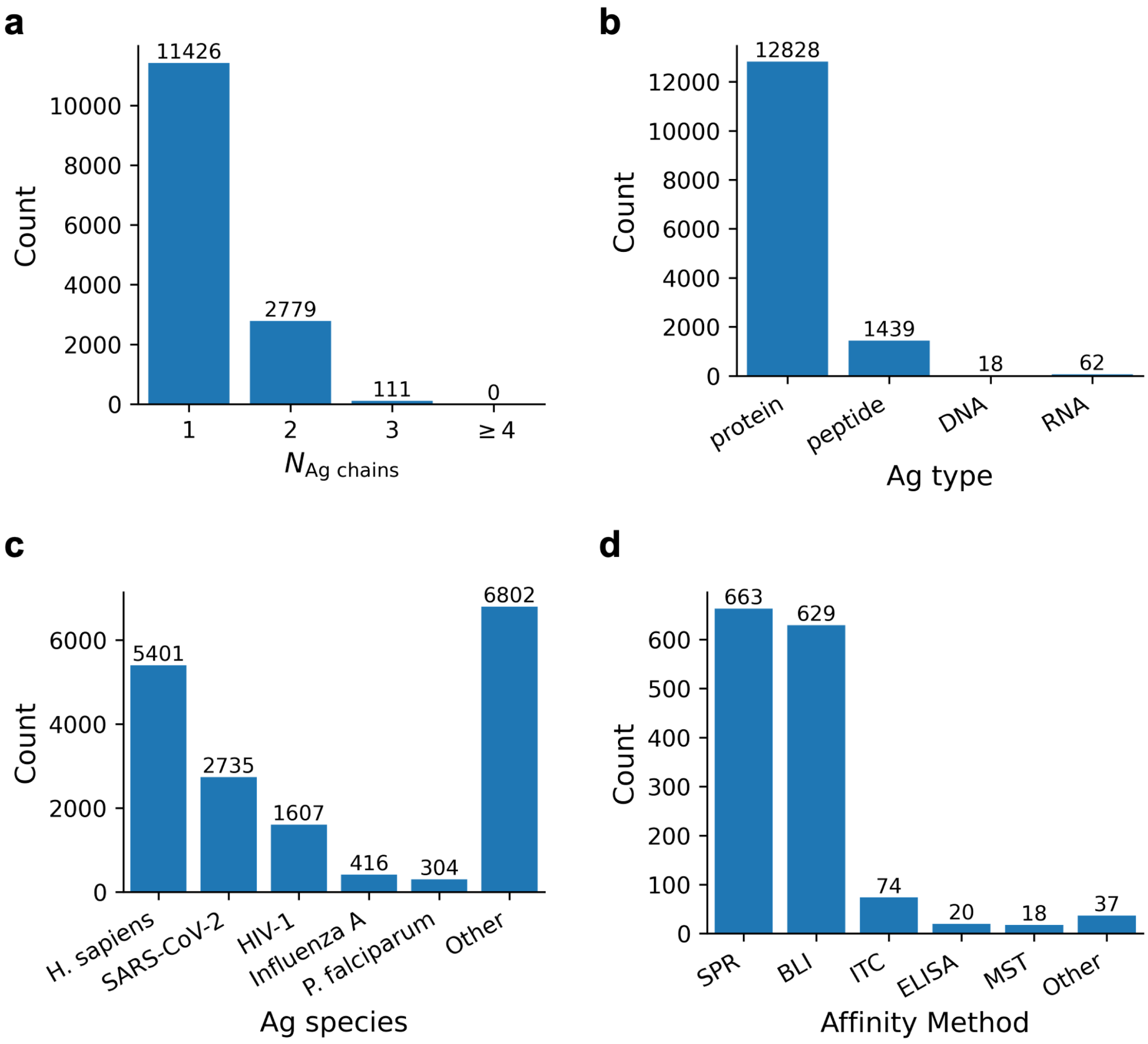

Supplementary Fig. 3. Additional statistics on antibody-antigen interactions (AAIs) in SAAINT-DB. a, Histogram of AAIs with varying numbers of antigen chains. **b**, Histogram of AAIs for different antigen types. **c**, Top sources of antigens. **d**, Top methods for antibody-antigen binding affinity measurement.

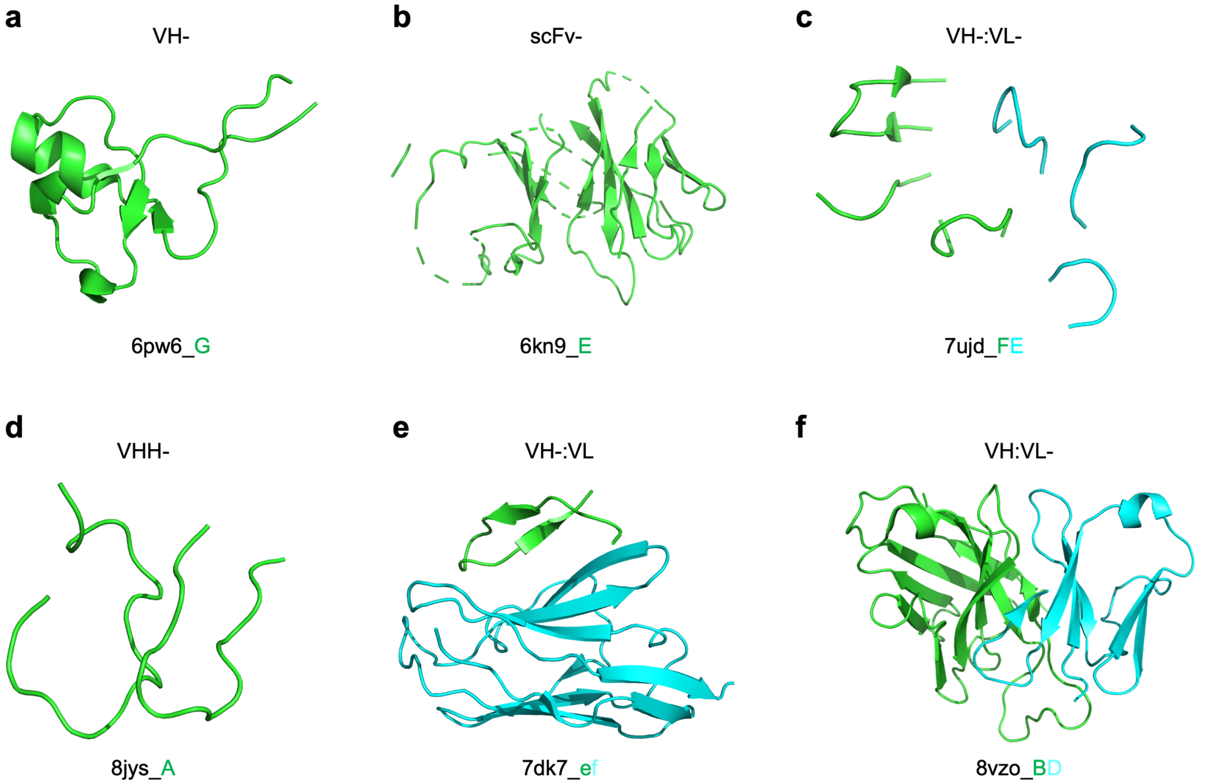

Supplementary Fig. 4. Example structures of antibody types with missing residues. Heavy and light chains are shown in green and cyan, respectively.

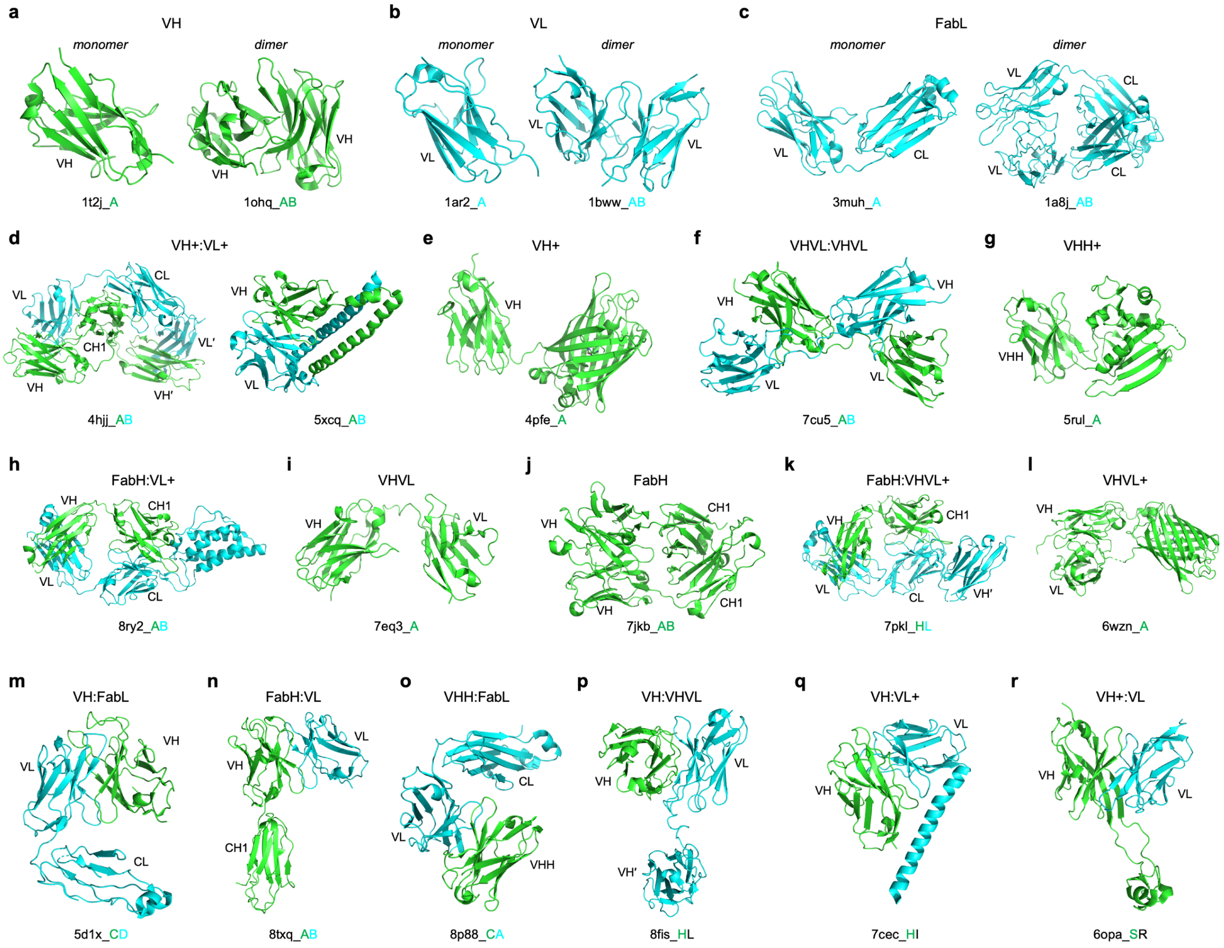

Supplementary Fig. 5. Example structures of other antibody types. Heavy and light chains are shown in green and cyan, respectively. Antibody domains, including VH, VL, CH1-3, and CL, are labeled for clarity.

Supplementary Table 1. Customized parameters for pairwise sequence alignment.

Parameter name	Value
alignment method	PairwiseAligner
mode	global
match_score	1
mismatch_score	-2
target_left_open_gap_score	0
target_left_extend_gap_score	0
target_internal_open_gap_score	-1
target_internal_extend_gap_score	0
target_right_open_gap_score	0
target_right_extend_gap_score	0
query_left_open_gap_score	-2
query_left_extend_gap_score	-2
query_internal_open_gap_score	-2
query_internal_extend_gap_score	-2
query_right_open_gap_score	-2
query_right_extend_gap_score	-2

Supplementary Table 2. List of SAAINT-parser parameters.

Task	Parameter name	Data type	Value
AbRSA type identification	min_len_ab_chain	int	60
	min_tmscore_ab_domain	float	0.4
Antigen type classification	max_len_pep	int	50
Ab type classification	min_len_vh	int	80
	max_len_vh	int	150
	min_len_vl	int	80
	max_len_vl	int	150
	min_len_fab	int	180
	max_len_fab	int	260
	min_len_vhvl	int	180
	max_len_vhvl	int	280
	max_radius_scfv	float	20.0
	max_pdb_seq_diff	int	60
HC-LC pairing	min_inf_res_num_vhm_vlm	int	8
	min_inf_res_num_vh_vl	int	20
	min_inf_res_num_fabh_vl	int	35
	min_inf_res_num_vh_fabl	int	35
	min_inf_res_num_fabh_fabl	int	35
	min_inf_res_num_vhvl_vhvl	int	80
	max_cdr_inf_res_ratio	float	0.7

Supplementary Table 3. List of SAAINT-DB data entry attributes.

Attribute	Explanation
PDB_ID	Four-letter PDB entry
Title	Summary title of the PDB entry
Mutation(s)	Indicates sequence mutations (value: yes/no)
Classification	Appropriate classification of the PDB entry
Deposit_date	PDB entry deposited date
Release_date	PDB entry released date
Method	Experimental method
Resolution	Average resolution of the PDB entry
R_free	R-Value Free
R_work	R-Value Work
PMID	PubMed ID of the PDB entry-associated primary publication
DOI	DOI of the PDB entry-associated primary publication
Model_index	Index of model within a PDB structure ensemble (from 0)
Asym_ID_type	PDB chain IDs assigned by the PDB (value: label_asym_id) or the deposition authors (value: auth_asym_id)
Ab_type	Antibody type (e.g., FabH:FabL, VH:VL, VHH, scFv, etc)
H_subgroup	Heavy chain V-gene subgroup
L_subgroup	Light chain V-gene subgroup
H_chain_ID	Heavy chain ID
L_chain_ID	Light chain ID
H_fas_seq	Heavy chain FASTA-seq
L_fas_seq	Light chain FASTA-seq
H_filled_pdb_seq	Heavy chain Filled-seq
L_filled_pdb_seq	Light chain Filled-seq
H_mean_radius	Mean radius of heavy chain structure
L_mean_radius	Mean radius of light chain structure
H_fas_seq_len	Length of heavy chain FASTA-seq
L_fas_seq_len	Length of light chain FASTA-seq
H_pdb_seq_len	Length of heavy chain PDB-seq
L_pdb_seq_len	Length of light chain PDB-seq
H_filled_seq_len	Length of heavy chain Filled-seq
L_filled_seq_len	Length of light chain Filled-seq
HL_inf_res_num	Number of interface residues of the HL pair
H_mol_name	Heavy chain molecule name
L_mol_name	Light chain molecule name
H_species	Heavy chain species
L_species	Light chain species
Ag_chain_ID(s)	Antigen chain ID(s)
Ag_type(s)	Antigen chain type(s) (value: protein, peptide, DNA, or RNA)
Ag_mol_name(s)	Antigen molecule name(s)
Ag_species	Antigen species
Ab_ag_inf_res_num	Number of Ab-Ag interface residues
CDR_inf_res_num	Number of CDR residues at Ab-Ag interface
CDR_inf_res_ratio	Ratio of interface CDR residues to the total interface Ab residues

Supplementary Table 4. List of 242 PDB entries recorded in SAAINT-DB but not in SAbDab.

1i3u, 2z93, 4hgm, 4xhj, 4xpg, 5b6f, 5bmf, 5fyj, 5fyk, 5kvl, 5ljy, 5nst, 6bjz, 6dzw, 6dzy, 6fs0, 6jmq, 6jmr, 6orv, 6pw6, 6rul, 6rum, 6s2i, 6uc5, 6w5a, 6w9g, 6wfw, 6wla, 7jic, 7jz1, 7jz4, 7k0z, 7k22, 7k23, 7m3l, 7mpg, 7n9u, 7n9x, 7qqb, 7qxc, 7qxd, 7qxe, 7sen, 7sgm, 7t6x, 7v3g, 7v3i, 7vh0, 8be3, 8be4, 8d53, 8e0g, 8ffj, 8fis, 8h3m, 8h3n, 8i4e, 8jru, 8jrv, 8p13, 8p15, 8pmw, 8pmy, 8q78, 8qp6, 8rrs, 8rrt, 8rru, 8rrv, 8rrw, 8rrx, 8rs0, 8s5z, 8s62, 8sal, 8sat, 8sw3, 8tb7, 8tgo, 8tgz, 8th3, 8tni, 8tox, 8tv5, 8txq, 8u9y, 8uls, 8vba, 8vbb, 8vgq, 8vh2, 8vve, 8vvg, 8w2e, 8wqw, 8x9u, 8xld, 8xvj, 8xvk, 8xvl, 8xxu, 8xxv, 8xyk, 8y0n, 8y0y, 8y3l, 8y3o, 8y3p, 8y3q, 8y3r, 8y3u, 8y45, 8y53, 8y63, 8y6w, 8ybs, 8yeg, 8yeh, 8yei, 8yfs, 8ygs, 8yn7, 8yn8, 8yna, 8yns, 8ynt, 8ywf, 8yyx, 8zhd, 8zhh, 8zhi, 8zhj, 8zhl, 8zhm, 8zl9, 8zp1, 8zp2, 8zpl, 8zpm, 8zyt, 8zyy, 9as0, 9as2, 9as6, 9asa, 9awx, 9awy, 9ax1, 9ax2, 9ax3, 9axp, 9b54, 9b6o, 9b6p, 9b6q, 9b6r, 9b6s, 9b7k, 9b7l, 9b7m, 9b7n, 9b7o, 9b7q, 9b7t, 9b7u, 9b7v, 9b7w, 9b7x, 9b9z, 9ba0, 9bde, 9bdt, 9bio, 9bp3, 9bq3, 9bqj, 9bub, 9buc, 9bud, 9c79, 9cgj, 9cgk, 9ci8, 9coo, 9csb, 9cxa, 9cz7, 9cza, 9czd, 9czf, 9d1w, 9d3e, 9d3g, 9dml, 9dqh, 9dqj, 9e60, 9e7w, 9e7x, 9e89, 9e8d, 9eah, 9eai, 9eaj, 9eru, 9eth, 9eti, 9exa, 9f33, 9f34, 9fxt, 9g6v, 9ge2, 9ge3, 9gwz, 9hvw, 9ik8, 9ik9, 9ipv, 9ivm, 9iya, 9iyb, 9iza, 9izd, 9izf, 9izg, 9j5v, 9jfu, 9jh3, 9jh6, 9kas, 9lld, 9lsh, 9lsi, 9lsj, 9m0d, 9mj4, 9mnw, 9mo0, 9mqi, 9mqk, 9mr7

Supplementary Table 5. List of 60 PDB entries with incorrectly paired heavy and light chains in SAbDab, correctly paired in SAAINT-DB.

PDB ID	SAAINT-DB HC-LC pairings	SAAINT-DB chain ID type	SAbDab HC-LC pairings	SAbDab chain ID type
1lmk	A:C, E:G	auth_asym_id	A:a, C:c, E:e, G:g	auth_asym_id
1moe	A:B	auth_asym_id	A:a, B:b	auth_asym_id
4hjj	H:L	auth_asym_id	H:N.A., N.A.:L	auth_asym_id
5fcs	H:L	auth_asym_id	H:h, L:l	auth_asym_id
5fhx	H:L	auth_asym_id	H:N.A., N.A.:L	auth_asym_id
5gru	H:L	auth_asym_id	H:h, L:l	auth_asym_id
5grw	A:B	auth_asym_id	A:a, B:b	auth_asym_id
5grx	G:H	auth_asym_id	G:g, H:h	auth_asym_id
5gry	A:G	auth_asym_id	A:a, G:g	auth_asym_id
5grz	A:G	auth_asym_id	A:a, G:g	auth_asym_id
5gs1	C:D, E:F, G:H, I:J, K:L, N:M, P:O, R:Q	auth_asym_id	C:c, D:d, E:e, F:f, G:g, H:h, I:i, J:j, K:k, L:l, N:M, P:O, R:Q	auth_asym_id
5gs2	D:H	auth_asym_id	D:d, H:h	auth_asym_id
5gs3	A:H	auth_asym_id	A:a, H:h	auth_asym_id
5hcg	H:L	auth_asym_id	H:N.A., N.A.:L	auth_asym_id
5iwl	A:B	auth_asym_id	A:a, B:b	auth_asym_id
5whz	H:L	auth_asym_id	H:N.A., N.A.:L	auth_asym_id
6ao0	H:L	auth_asym_id	H:N.A., N.A.:L	auth_asym_id
6hjq	G:H, I:J, K:L	auth_asym_id	I:J, K:L	auth_asym_id
6kr0	A:B, C:D	auth_asym_id	A:a, B:b, C:c, D:d	auth_asym_id
6o89	H:L	auth_asym_id	H:N.A., N.A.:L	auth_asym_id
6o8d	H:L	auth_asym_id	H:N.A., N.A.:L	auth_asym_id
6qkd	H:L, I:T, J:M, K:O	auth_asym_id	H:N.A., I:N.A., J:N.A., K:N.A., N.A.:L, N.A.:M, N.A.:O, N.A.:T	auth_asym_id
6vrp	A:B	auth_asym_id	A:a, B:b	auth_asym_id
6vun	A:B	auth_asym_id	A:a, B:b	auth_asym_id
6vuo	A:B	auth_asym_id	A:a, B:b	auth_asym_id
6x9x	A:B	auth_asym_id	A:N.A., N.A.:B	auth_asym_id
6ymq	A:N.A., B:N.A., C:N.A., D:N.A., L:N.A., K:N.A.	label_asym_id	A:a, B:b, C:c, E:e, F:f	auth_asym_id
6yz7	B:C, G:H, D:N.A., F:N.A.	label_asym_id	D:N.A.	auth_asym_id
6zqk	B:A, D:C	auth_asym_id	A:N.A., B:N.A., C:N.A., D:N.A.	auth_asym_id
7cu5	A:B	auth_asym_id	A:a, B:b	auth_asym_id
7joo	H:L	auth_asym_id	H:L, K:N.A.	auth_asym_id
7mlv	L:M, G:J, H:I, F:K	auth_asym_id	L:M, G:J, H:I, F:N.A., N.A.:K	auth_asym_id
7pkl	H:L	auth_asym_id	H:N.A., L:l	auth_asym_id
7sn3	D:E, F:G, H:L	auth_asym_id	D:N.A., N.A.:E, F:G, H:L	auth_asym_id
7t0w	H:F, G:I	label_asym_id	H:L	auth_asym_id
7t1w	A:B, C:D, E:F, G:H, I:J	label_asym_id	H:L	auth_asym_id
7t1x	A:B, C:D, E:F, G:H, I:J	label_asym_id	H:L	auth_asym_id
7uih	D:C, F:E	auth_asym_id	D:N.A., F:N.A., N.A.:C, N.A.:E	auth_asym_id
7ujd	D:C, F:E	auth_asym_id	D:N.A., F:N.A., N.A.:C, N.A.:E	auth_asym_id
7uyl	H:L	auth_asym_id	H:L, K:N.A.	auth_asym_id
7uym	H:L	auth_asym_id	H:L, K:N.A.	auth_asym_id
7xod	X:Y, R:S, U:V, T:N.A.,	auth_asym_id	X:Y, R:N.A., N.A.:S, U:V, T:N.A.,	auth_asym_id

	W:N.A., Z:N.A.		W:N.A., Z:N.A.	
7yc5	H:E, G:D, I:F	auth_asym_id	D:N.A., E:N.A., F:N.A., G:N.A., H:N.A., I:N.A.	auth_asym_id
7ye5	H:L, G:I	auth_asym_id	H:L	auth_asym_id
7yvn	L:K, I:H, O:N	auth_asym_id	L:K, I:N.A., N.A.:H, O:N	auth_asym_id
7yvp	I:J, K:H, L:M, P:O	auth_asym_id	I:N.A., K:H, N.A.:J, L:M, P:O	auth_asym_id
8blq	A:B, E:C	auth_asym_id	E:C, A:N.A., N.A.:B	auth_asym_id
8c7j	A:B	auth_asym_id	A:N.A., N.A.:B	auth_asym_id
8dxs	H:L, G:J, F:N.A., N.A.:I	auth_asym_id	H:L, F:N.A., G:N.A., N.A.:I, N.A.:J	auth_asym_id
8hhx	F:G, H:I	auth_asym_id	F:G, H:N.A., N.A.:I	auth_asym_id
8hhy	H:L, F:W, G:I	auth_asym_id	H:L, F:N.A., G:N.A., N.A.:I, N.A.:W	auth_asym_id
8jf7	F:E, J:K, H:G	auth_asym_id	J:K, F:N.A., H:N.A., N.A.:E, N.A.:G	auth_asym_id
8r3w	A:R	auth_asym_id	A:a, R:r	auth_asym_id
8r40	A:R	auth_asym_id	A:a, R:r	auth_asym_id
8u2c	D:C, F:E	auth_asym_id	D:C, F:E, I:N.A., J:N.A., N.A.:K, N.A.:L	auth_asym_id
8vtr	A:G	auth_asym_id	A:N.A., N.A.:G	auth_asym_id
8vu1	A:G	auth_asym_id	A:N.A., N.A.:G	auth_asym_id
8vua	A:G	auth_asym_id	A:N.A., N.A.:G	auth_asym_id
8xbf	D:E, F:G	auth_asym_id	D:E, F:N.A., N.A.:G	auth_asym_id
9dzq	G:I, H:J, C:E, D:F	auth_asym_id	G:I, H:J, C:N.A., D:N.A., N.A.:E, N.A.:F	auth_asym_id

Notes: (1) SAAINT-parser automatically determined whether to use auth_asym_id or label_asym_id chain IDs. (2) N.A. stands for no pairing chain ID. (3) The only less accurate pairing in SAAINT-DB was 8dxs, in which chains F and I might form a VH:VL pair through visual inspection. However, chains F and I were distinct from each other, resulting in only nine residues at the interface. In comparsion, the same Ab chain pairs H:L and G:J had 36 and 27 residues at their respective interfaces.

Supplementary Table 6. Summary of methodological similarity and key differences among SAAINT-DB, SAbDab, and AbDab.

<table>
<tr><td>Similarity</td><td>Three databases—SAAINT-DB, SAbDab, and AbDb—were constructed using similar generl steps: Ab chain identification, HC-LC pairing, and Ab-Ag pairing.</td></tr>
<tr><td>Key differences</td><td>The specific methods used at each step differ:
1. <u>Ab chain identification</u>
• SAAINT-DB uses AbRSA to label chains as: HC, LC, HLC (single chain containing both variable regions), or non-Ab.
• SAbDab uses AbNum to classify chains as HC, LC, or non-Ab.
• AbDb uses idabchain to label chains as HC, LC, or Ag.
2. <u>Ab type classification</u>
• SAAINT-DB categorizes Abs into 29 distinct types, such as FabH:FabL, VH:VL, VHH, scFv, etc.
• SAbDab classifies Abs simply as either scFv or non-scFv.
• AbDb assigns Abs to one of three types: complete (paired HC and LC), LC-only, or HC-only.
3. <u>HC-LC pairing</u>
• SAAINT-DB pairs HCs and LCs using a two-step approach:
1. Apply variable cutoffs to the number of HC-LC interface residues ($N_{HL\_inf\_res}$) to exclude false pairings.
2. Use a combination of greedy search and iterative heuristic search, guided by a custom score function, to maximize valid HL pairs across the structure.
• SAbDab pairs HCs and LCs by requiring that the conserved cystenine at Chothia position 92 on HC is within 22 Å of the conserved cystenine at position 88 on LC.
• AbDb handles each Ab type differently:
1. For HC-only Abs: no pairing is performed.
2. For LC-only Abs: two LCs are considered paired if the Ca atoms of two L36 residues (Kabat numbering) are within 20 Å, and the Ca atoms of L87 residues are also within 20 Å.
3. For complete Abs: HL pairs are identified by maximizing the number of contacts, defined as atom centers within 4 Å of each other.
4. Ab-Ag pairing
• SAAINT-DB uses the same approach as HC-LC pairing, with three conditions:
1. Number of residues at the Ab-Ag interface ($N_{ab\_ag\_inf\_res}$) ≥ 10
2. Number of CDR residues at the Ab-Ag interface ($N_{CDR\_inf\_res}$) ≥ 5
3. Ratio of CDR interface residue to total interface residues ($R_{CDR\_inf\_res}$) ≥ 0.25
• SAbDab pairs Abs with Ags by counting CDR residues within 7.5 Å of each Ag chain candidate.
• AbDb categorizes Ags into two groups:
1. HETATM non-protein Ags (e.g., haptens, lipids and carbohydrates): considered as Ag if any atom pairs are within 4 Å, provided no CONECT records suggest covalent binding with Ab.
2. protein/nucleotide Ags: identified as Ags if (1) more contacts are made with CDRs than with framework, and (2) there are at least 15 contacts with CDRs.</td></tr>
</table>
